# Anxiety, Motivation, and Competence in Mathematics and Reading for Children With and Without Learning Difficulties

**DOI:** 10.3389/fpsyg.2021.704821

**Published:** 2021-10-07

**Authors:** Courtney Pollack, Dayna Wilmot, Tracy M. Centanni, Kelly Halverson, Isabelle Frosch, Anila M. D'Mello, Rachel R. Romeo, Andrea Imhof, Jimmy Capella, Karolina Wade, Noor Z. Al Dahhan, John D. E. Gabrieli, Joanna A. Christodoulou

**Affiliations:** ^1^Department of Brain and Cognitive Sciences & McGovern Institute for Brain Research, Massachusetts Institute of Technology, Cambridge, MA, United States; ^2^Department of Psychology, Texas Christian University, Fort Worth, TX, United States; ^3^Department of Clinical Psychology, University of Houston, Houston, TX, United States; ^4^Department of Psychology, Harvard University, Cambridge, MA, United States; ^5^Department of Clinical Psychology, University of Oregon, Eugene, OR, United States; ^6^Department of Communication Sciences and Disorders, MGH Institute of Health Professions, Boston, MA, United States

**Keywords:** mathematics, reading, anxiety, motivation, competence, learning difficulties

## Abstract

Knowledge of the relations among learners' socio-emotional characteristics and competencies as they engage in mathematics and reading is limited, especially for children with academic difficulties. This study examined the relations between anxiety, motivation, and competence in mathematics and reading, within and across domains, in an academically-diverse set of 8–13-year-old learners (*n* = 146). To measure anxiety and motivation across domains, we paired existing measures of math anxiety and reading motivation with researcher-developed analogs for reading anxiety and math motivation. Participants completed standardized assessments of mathematics and reading, anxiety and motivation surveys for math and reading, and a measure of nonverbal cognitive ability. Results showed high internal consistency for all anxiety and motivation scales (Cronbach's alpha = 0.76–0.91). Pearson correlations showed that within and across domains, participants with higher competence had lower anxiety and higher motivation. Higher anxiety was also associated with lower motivation. Regression analyses showed that for both math and reading, within-domain motivation was a stronger predictor of competence than anxiety. There was a unidirectional across-domain relation: socio-emotional characteristics for reading predicted math competence, after accounting for nonverbal cognitive ability, age, gender, and within-domain anxiety and motivation. Results contribute to knowledge of the socio-emotional characteristics of children with and without learning difficulties in association with reading and math activities. Implications of a unidirectional socio-emotional link between the two domains can advance research and theory of the relations among socio-emotional characteristics and competence for academically-diverse learners.

## Introduction

Socio-emotional characteristics, such as anxiety and motivation, are important for schooling and beyond. As examples, learners with high levels of math anxiety may avoid math during schooling (Hembree, 1990), postsecondary education, and career selection (e.g., Ashcraft, 2002). Learners with higher motivation to read tend to have higher reading performance in middle grades and secondary school (Retelsdorf et al., 2011; Froiland and Oros, 2014). Whether socio-emotional characteristics affect skill development across domains is also important, given that academic domains, such as math and reading, are interrelated (e.g., Duncan et al., 2007; Vanbinst et al., 2020b). Knowledge of the relations between anxiety, motivation, and competence in math and reading within and across domains is limited, especially for children who struggle in math, reading, or both. Such knowledge can illuminate important contextual factors for learners across domains, to help reduce barriers to learning, and to identify potential mechanisms of resilience. In this study, we examine elementary school children's anxiety, motivation, and competence within and across math and reading for those with and without learning disabilities.

The most well-studied socio-emotional construct in math is anxiety. Math anxiety is domain-specific apprehension, fear, or worry when engaging with math content (e.g., Ashcraft, 2002; Dowker, 2019a). Math anxiety manifests physiologically (Dowker et al., 2016; Suárez-Pellicioni et al., 2016; Ramirez et al., 2018), can be transmitted intergenerationally (Vanbinst et al., 2020a) and in the classroom (Beilock et al., 2010), and is higher in girls than in boys, on average, in as early as primary school (Dowker et al., 2016; Hill et al., 2016). There is a well-established link between higher levels of math anxiety and lower math competence across childhood, adolescence, and adulthood (Dowker et al., 2016; Foley et al., 2017; Dowker, 2019a) that begins in the early grades (Ma, 1999; Wu et al., 2012; Barroso et al., 2021; Szczygieł and Pieronkiewicz, 2021). This relation between math anxiety and math competence also holds for children with math disability, which is a difficulty in arithmetic and numerosity processing (Rubinsten and Tannock, 2010). While children with math disability may be more likely to have high math anxiety, most math anxious individuals are typically- or high-achieving (Devine et al., 2018), which underscores the importance of understanding math anxiety across a range of learners.

Compared to math anxiety, other socio-emotional characteristics of math, like motivation, have received less attention (see Dowker, 2019a). One reason may be that people may be more anxious about math than other subjects (Punaro and Reeve, 2012; Dowker et al., 2016; Dowker, 2019b). Math motivation has been operationalized in myriad and partially-overlapping ways, such as interest, engagement, enjoyment, self-perceived abilities, and self-efficacy (Kriegbaum et al., 2015; Baten et al., 2019). Generally, higher math motivation or attitudes toward math have been associated with lower math anxiety (Hembree, 1990; Zakaria and Nordin, 2008; Jain and Dowson, 2009; Jameson, 2014; Luttenberger et al., 2018). Math anxiety and positive attitudes may show an inverse relation generally, but they are not opposite ends of the same spectrum. One framework offers that math attitudes can be considered a cognitive factor, while math anxiety can be considered an emotional factor (Dowker et al., 2016). In other words, positive attitudes are not the mere absence of anxiety. For instance, research has shown positive relations between math attitudes and math achievement that persist when controlling for anxiety (Villavicencio and Bernardo, as cited in Dowker, 2019b). Higher math motivation or attitudes toward math are also associated with higher math competence (Zakaria and Nordin, 2008; Krinzinger et al., 2009; Seaton et al., 2014; Kriegbaum et al., 2015; Arens et al., 2017; Lohbeck, 2018) in adults and children (but see Wang et al., 2015). Math motivation may mediate the relation between math anxiety and competence (Justicia-Galiano et al., 2017). Math anxiety and motivation may be reciprocally related across time (e.g., Gunderson et al., 2018). Additional research is needed to inform how math anxiety and motivation relate to math competence across children with and without learning disabilities.

The reading domain has an opposite story: a growing but limited body of literature on reading anxiety and a more developed body of literature on reading motivation. Reading anxiety—negative emotional, cognitive, and physiological reactions to reading (Jalongo and Hirsh, 2010; Piccolo et al., 2017)—has received little attention (Piccolo et al., 2017). Prior research has mostly focused on relations between reading and general or trait anxiety, with higher levels of anxiety among adults and children with reading disabilities compared to typical readers (Casey et al., 1992; Carroll et al., 2005; Carroll and Iles, 2006; Grills-Taquechel et al., 2012; Grills et al., 2014; Elgendi et al., 2021; Hossain et al., 2021). Other socio-emotional characteristics of reading, including motivation, have received comparatively more attention. Reading motivation has been conceptualized in various ways such as self-concept; beliefs about reading, reading attitudes, or interest (see Conradi et al., 2014); or engagement and persistence (Urdan and Schoenfelder, 2006). Generally, higher reading motivation has been associated with better reading competence (e.g., Chapman and Tunmer, 2003). Reading attitudes and perceptions have been positively associated with reading skills in adolescents (Conlon et al., 2006) and higher self-concept has been associated with higher reading competence for children (Chapman and Tunmer, 1995).

With so few studies on reading anxiety, knowledge of the relations among reading anxiety, motivation, and competence is limited, but emerging. Katzir et al. (2018) examined the relations among reading anxiety, reading self-concept, and reading competence in 7–9-year-old Israeli children. The authors found that higher reading anxiety was associated with lower reading self-concept. They also found differences by gender, in which girls had higher reading anxiety and lower reading self-concept than boys, despite having higher reading accuracy. In another study, Ramirez et al. (2019) examined the relations among reading anxiety, reading affect (i.e., enjoyment), and reading competence for first and second grade U.S. children (roughly ages 6–8). They found that higher levels of reading anxiety were associated with lower reading competence, on average, and that reading anxiety was more strongly related to reading competence than positive reading affect. In contrast to Katzir et al. (2018), Ramirez et al. (2019) found that boys were more susceptible to the effects of reading anxiety compared to girls. Scale, construct, and cross-cultural differences may contribute to a lack of convergence of findings across studies. Together, these studies illustrate that relations among reading anxiety, motivation, and competence in children need further examination.

Beyond further clarification of the relations between socio-emotional characteristics and competence within domain, the interrelation of math and reading suggests the need for research across domains. Math and reading skills are already interrelated for young children. Vanbinst et al. (2020b) found that phonological awareness and numeral recognition correlated with both early arithmetic and early reading skills in 5-year-old children. The authors concluded that phonological awareness and numeral recognition were shared cognitive correlates of math and reading. Cui et al. (2019) found that visual form perception of geometric shapes related to both reading and arithmetic skills in elementary school children. Neuroimaging research suggests shared functional neural correlates for arithmetic and phonological processing in children (Pollack and Ashby, 2018; Kersey et al., 2019). This cross-domain relation between math and reading also holds for children with learning disabilities. Children with reading disabilities (e.g., dyslexia) struggle with aspects of math, especially arithmetic fact fluency (Simmons and Singleton, 2008; Boets and De Smedt, 2010; De Smedt and Boets, 2010; Vukovic et al., 2010; Evans et al., 2014; Koerte et al., 2016). Even with a normal range of math performance, children with dyslexia are less accurate and slower with fact retrieval than their typically-developing peers (Boets and De Smedt, 2010). Added to these interrelations is a substantial comorbidity of math and reading learning disabilities (Barbaresi et al., 2005; Kovas et al., 2007; Dirks et al., 2008; Landerl and Moll, 2010). These interrelations suggest that socio-emotional characteristics in one domain may relate to competence in another, especially across academically-diverse learners.

The mechanisms through which domain-specific anxiety, motivation, and competence affect each other are not fully understood. Experiences doing math may affect socio-emotional characteristics toward math, which in turn may affect subsequent math experiences (e.g., Jansen et al., 2013; Dowker et al., 2016). Alternatively, higher anxiety in math may lead to math avoidance or reduced working memory, either of which may lead to lower math performance (for reviews see Carey et al., 2016; Dowker et al., 2016; Dowker, 2019b). Or, these relations may be bidirectional over time (e.g., Carey et al., 2016). The same potential mechanisms may operate in the reading domain (e.g., Katzir et al., 2018). We speculate that these mechanisms may also apply across math and reading for academically-diverse learners due to the relation of skills across domains. For instance, children who struggle with reading may have higher math anxiety and/or lower math motivation, which could be because phonological processing is related to math fact retrieval and because reading skills are used in other areas of math, like reading word problems.

In sum, within-domain relations among anxiety, motivation, and competence in math and in reading are already present for children in elementary (or primary) school. Yet, there are substantial differences in knowledge of the within-domain relations between socio-emotional characteristics and competence in math and reading across a range of learners. These differences make it difficult to understand their interrelation, particularly in young learners who may have math or reading disabilities, or both. Further, to the best of our knowledge, there are no existing studies focused on the relations among these socio-emotional characteristics and competencies across domains in academically-diverse learners.

We address these gaps by examining the relations among anxiety, motivation, and competence across math and reading for children with and without learning disabilities in math, reading, or both. To evaluate comparable factors in both reading and math in the same sample, we developed analogs to existing scales to create pairs of parallel measures for anxiety and motivation in math and reading. We then administered standardized measures of math and reading and the anxiety and motivation scales to an academically-diverse sample of children. We used multiple regression to examine whether socio-emotional characteristics within and across domain predicted academic competence and whether these relations persisted when controlling for nonverbal cognitive ability, age, and gender. We hypothesized that there would be relations among anxiety, motivation, and competence within each domain that would persist after controlling for nonverbal cognitive ability, age, and gender. Based on the interrelation of math and reading skills, we hypothesized that anxiety and motivation would relate to competence across domains, though within-domain relations would be stronger.

## Materials and Methods

### Participants

Participants were 146 academically-diverse children 8–13 years old (*M* = 10.8, *SD* = 1.1; 47% male) who in the U.S were part of a larger study on math and reading disabilities. As part of the larger study, we used purposeful recruiting to seek an overrepresentation of children with learning disabilities compared to the general population (see section Group Characterizations). We wanted to examine the relations among anxiety, motivation, and competence within and across domains for the full range of learners. That is, we were interested in whether relations would apply across a large performance spectrum, with children who are lower performers, average performers, and higher performers across math and reading. With a sample of about 145, a representative sample of 15% with learning disabilities would result in only about 20 children, which seemed to us to be too small to examine the full range of achievement across both math and reading. Participants' racial and ethnic identities, based on the U.S. Census categories, were 73% White, 6% Asian, 4% Black/African American, 1% Hispanic/Latino, 13% more than one race, and 3% undisclosed.

To the best of our knowledge, there is a dearth of studies that simultaneously examine the relations of anxiety and motivation with competence across domain, which precluded an *a priori* power analysis based on existing effect sizes. Related studies on the relations between socio-emotional characteristics and competence within math and reading domains had sample sizes ranging from 115 to 167 (Krinzinger et al., 2009; Justicia-Galiano et al., 2017; Katzir et al., 2018), suggesting that the sample size of the present study was generally in line with prior research.

Participants were recruited through flyers in the community, online posting, a database of participants from prior studies, and through cross-promotion with other studies. The Committee on the Use of Humans as Experimental Subjects (COUHES) at the Massachusetts Institute of Technology approved the study. Parents or guardians provided consent and children provided assent to participate.

### Measures

Participants completed a comprehensive battery of language, reading, math, cognitive, and socio-emotional assessments as part of the larger study. The present study includes socio-emotional measures in math and reading, standardized assessments of math and reading, and a measure of nonverbal cognitive ability.

#### Socio-Emotional Measures

##### Anxiety

Participants completed the Math Anxiety Scale for Young Children, Revised (i.e., MASYC-R; Ganley and McGraw, 2016). The MASYC-R is a 13-item scale that measures math anxiety overall and on three subscales: negative reactions (items 1–4), confidence (items 5–7), and worry (items 8–13). To measure reading anxiety, we created the Reading Anxiety Scale for Young Children (i.e., RASYC) using Ganley and McGraw's (2016) MASYC-R. To create the RASYC, we modified item language to reflect reading anxiety. As examples, we changed “Math gives me a stomach ache” to “Reading gives me a stomach ache” and “I like to raise my hand in math class” to “I like to raise my hand in reading/English class.” Importantly, the math anxiety scale did not include questions that involved reading and the reading anxiety scale did not include questions that involved math. For example, the math anxiety scale did not include any questions about word problems. Scoring for the RASYC followed Ganley and McGraw's (2016) scoring for the MASYC-R. Scores for the negative reactions and worry subscales were scored as Yes = 4, Sometimes = 3, Not really = 2, No = 1. Confidence subscale items have reverse scoring (e.g., Yes = 1), such that a higher score is associated with lower confidence and for overall scores, a higher score is associated with greater anxiety.

##### Motivation

Participants completed the Motivation to Read Profile-Revised (i.e., MRP-R; Malloy et al., 2013). The MRP-R is a 20-item scale that measures motivation to read. The survey contains two subscales: self-concept (odd-numbered items) and value (even-numbered items). Each item has four answer choices (scored 1–4), with higher scores representing higher self-concept or value. Prior studies have operationalized math motivation in varied ways. In line with conceptualizations of reading motivation (Urdan and Schoenfelder, 2006; Malloy et al., 2013), we define math motivation as the willingness for children to engage and persist with math, measured by children's value of math and self-concept in math. To measure motivation, we created the Motivation for Math Profile (i.e., MMP) using Malloy et al.'s (2013) MRP-R. We modified item wording to reflect math instead of reading. As an example, for the self-concept item “My friends think I am ____” with response options of “a very good reader; a good reader; an OK reader; a poor reader,” we changed the answer choices to “very good at math; good at math; OK at math; bad at math.” As an example of a value item, we changed the stem “Reading is something I like to do” to “Doing math problems is something I like to do.” The math motivation scale did not include questions that involved reading and the reading motivation scale did not include questions that involved math. Scoring for the MMP followed Malloy et al.'s (2013) scoring guide (p. 279) for overall motivation, and subscale scores for self-concept and value.

To standardize administration across children of different reading levels, we administered the scales orally in a quiet location. A researcher read each item stem and answer choices aloud to the child, while the researcher and child both looked at the scale on a computer screen. The researcher selected each answer that the child chose.

#### Math and Reading Competence

We measured mathematical competence with two composites. The Broad Mathematics composite of the Woodcock Johnson-IV (Schrank et al., 2014) includes three subtests. Math Fluency is a timed 3-min test of addition, subtraction, and multiplication fact fluency. The Calculation subtest is an untimed written test of calculation problems from single-digit arithmetic through calculus. The Applied Problems subtest is an untimed test in which participants analyze and solve math problems. The Math Fluency composite of the Wechsler Individual Achievement Test-III (WIAT-III, Psychological Corporation, 2009) measures fact fluency with separate 1-min timed addition, subtraction, and multiplication tests.

We measured reading competence with two composites. The Total Word Reading Efficiency composite of the TOWRE-2 (Torgesen et al., 2012) is comprised of timed measures of sight word reading and pseudoword reading. The Basic Skills composite of the WRMT-III (Woodcock, 2011) includes untimed measures of word reading (Word Identification) and pseudoword reading (Word Attack). Analyses include age-adjusted standard scores for all competence measures (based on a mean of 100 and a standard deviation of 15).

#### Nonverbal Cognitive Ability

We measured nonverbal cognitive ability using the Kaufman Brief Intelligence Test (KBIT-2; Kaufman and Kaufman, 2004) Matrices subtest, in which participants select which image fits into a matrix. We used a measure of nonverbal cognitive ability because scores on measures of verbal cognitive ability may be artificially lower for children with reading disability due to differences in exposure and background knowledge related to reading. To be included in the study, participants had to have a standard score of 80 or greater. Analyses include standard scores.

### Analyses

#### Group Characterizations

To examine whether the sample was academically diverse with a relatively high prevalence of children with learning disabilities, we screened participants for having math and/or reading disability using the standard math and reading competence measures. Participants in the math disability only group had a history or diagnosis of math disability, scored below 90 on at least two of the math subtests, and scored at or above 90 on all reading subtests. Participants in the reading disability only group had a history or diagnosis of reading disability, scored below 90 on at least two of the reading subtests, and scored at or about 90 on all math subtests. Participants in the comorbid math and reading disability group had some history of math and reading disability, and scored below 90 on at least two math subtests and at least two reading subtests.

We also characterized participants without learning disabilities. These participants had no personal or family history of math or reading disability. They had standard scores at or above 90 on all math and reading subtests. Participants who did not fit any set of criteria did not belong to a group. As we show in section Group Characterizations: Incidence of Learning Disabilities below, group characterizations revealed sample sizes that were too small for group comparisons. Therefore, all analyses used a multiple regression approach with the full sample as we describe in section Socio-Emotional Measures and Competence Within and Across Domains.

#### Socio-Emotional Measures and Competence Within and Across Domains

To examine reliability of the new and existing scales, we calculated Cronbach's alpha for each full scale and all subscales.

We used multiple regression to examine whether socio-emotional measures predicted competence within and across domain, while accounting for nonverbal cognitive ability, age, and gender. Because our research questions focus on anxiety and motivation, rather than specific aspects like value or worry, and due to the number of subscales across the four outcomes and four measures, analyses include full scale scores for socio-emotional and competence measures.

Equation (1) describes the model:


(1) $$\begin{array}{r} Y_{i}=\beta_{0}+\beta_{1}A_{1i}+\beta_{2}A_{2i}+\beta_{3}M_{1i}+\beta_{4}M_{2i}+\beta_{5}X_{i}+e_{i} \end{array}$$

In Equation (1), *Y*_*i*_ refers to each outcome (i.e., Broad Mathematics, Math Fluency, Total Word Reading Efficiency, Basic Skills) for each participant *i*. *A*_1*i*_ refers to the within-domain anxiety scale associated with *Y*_*i*_. *A*_2*i*_ refers to the across-domain anxiety scale for outcome *Y*_*i*_ for participant *i*. *M*_1*i*_ refers to the within-domain motivation scale associated with *Y*_*i*_ and *M*_2*i*_ refers to the across-domain motivation scale. ***X***_*i*_ refers to a set of three covariates that include nonverbal cognitive ability, age, and gender for each participant *i*. We individually include standard scores for nonverbal cognitive ability, age in years, and a dichotomous variable (1 = Boy) for gender. For each outcome, we fit a taxonomy of models in which we sequentially add predictors as Equation (1) specifies.

## Results

### Group Characterizations: Incidence of Learning Disabilities

Group characterizations show that the sample was academically diverse, with 34% (49/146) of participants having a learning disability. Three participants met the criteria for math-only disability. Thirteen participants met the criteria for reading-only disability and 33 participants met the criteria for comorbid math and reading disability. Sixty-eight participants met criteria for having no learning disability and the remaining 29 did not have a group. These participants had heterogeneous score patterns and may have, for example, scored below the cutoff for only one of the measures in one or both domains and may or may not have had a history of learning disabilities. Supplementary Table 1 shows age and performance on the competence and socio-emotional measures by group, excluding the math disability only group due to small sample size. Due to the small sample sizes by group, we are unable to conduct group comparisons. Instead, we provide descriptive statistics to illustrate the academically-diverse nature of the sample.

### Preliminary Analyses

Table 1 presents descriptive statistics for competence and socio-emotional measures for each domain, for the full sample (*n* = 146). Table 2 presents Cronbach's alpha for each full scale and subscale for the four socio-emotional measures. Scales showed good to high internal consistency (α = 0.76–0.91).

**Table 1:** Descriptive statistics for reading and mathematics competence, and reading and mathematics motivation and anxiety (*n* = 146).

**Measure**	**Mean**	**SD**	**Minimum**	**Maximum**
**Math competence**
Broad mathematics	101.15	16.82	58	139
Math fluency	98.92	16.61	62	142
**Reading competence**
Total Word Reading Efficiency	97.47	16.39	58	130
Basic Skills	97.86	17.00	55	136
**Anxiety and motivation**
Math anxiety	23.60	7.74	13	49
Math motivation	57.83	9.97	29	77
Reading anxiety	22.15	6.67	13	44
Reading motivation	58.64	9.16	29	76
Nonverbal cognitive ability	112.30	14.09	82	143

**Table 2:** Cronbach's alpha for each full scale and subscale (*n* = 146).

	**Construct**	
	**Math Anxiety^b^**	**Reading Anxiety^a^**
	**(MASYC-R)**	**(RASYC)**
Full scale	0.88	0.84
Negative reactions	0.69	0.73
Confidence	0.85	0.83
Worry	0.83	0.76
	**Motivation for Math^a^**	**Motivation to Read^b^**
	**(MMP)**	**(MRP-R)**
Full scale	0.91	0.89
Self-confidence	0.89	0.82
Value	0.87	0.84

a *New scale;*

b *Existing scale*.

In Table 3, we present bivariate correlations and significance levels among competence, anxiety, and motivation measures for math and reading, nonverbal cognitive ability, and age. As the table shows, competence measures had strong, positive, statistically significant correlations within domain and across domains. Competence was correlated with socio-emotional measures within and across domains. Higher math competence was associated with lower math anxiety and higher math motivation; both correlations were statistically significant and moderate. Across domains, higher math competence was associated with higher reading motivation and lower reading anxiety; all correlations were statistically significant and were small-to-moderate. Similarly, reading competence had a moderate positive correlation with reading motivation and moderate negative association with reading anxiety, and both were statistically significant. Across domains, higher reading competence was associated with lower math anxiety and higher math motivation. Correlations were small and statistically significant.

**Table 3:** Bivariate correlations among competence (1–4), socio-emotional characteristics (5–8), KBIT scores, and age (*n* = 146).

	**(1)**	**(2)**	**(3)**	**(4)**	**(5)**	**(6)**	**(7)**	**(8)**	**(9)**	**(10)**
(1) Broad Math	1.000									
(2) Math Fluency	0.916^***^	1.000								
(3) Total Word Reading Efficiency	0.711^***^	0.682^***^	1.000							
(4) Basic Skills	0.648^***^	0.620^***^	0.890^***^	1.000						
(5) Math anxiety	−0.423^***^	−0.408^***^	−0.211^*^	−0.216^**^	1.000					
(6) Math motivation	0.533^***^	0.490^***^	0.285^***^	0.252^**^	−0.711^***^	1.000				
(7) Reading anxiety	−0.387^***^	−0.357^***^	−0.495^***^	−0.452^***^	0.439^***^	−0.297^***^	1.000			
(8) Reading motivation	0.392^***^	0.326^***^	0.608^***^	0.546^***^	−0.184^*^	0.316^***^	−0.652^***^	1.000		
(9) Nonverbal cognitive ability	0.606^***^	0.500^***^	0.498^***^	0.497^***^	−0.271^**^	0.310^***^	−0.251^**^	0.301^***^	1.000	
(10) Age	−0.330^***^	−0.351^***^	−0.397^***^	−0.459^***^	0.178^*^	−0.156^~^	0.259^**^	−0.295^***^	−0.406^***^	1.000

*~p = 0.06*,

* *p < 0.05*,

** *p < 0.01, and*

*** *p < 0.001*.

As Table 3 shows, all socio-emotional measures were statistically significantly correlated with one another. Correlations were strong and negative between anxiety and motivation within domain. Across domain, higher math anxiety was associated with higher reading anxiety and higher math motivation was associated with higher reading motivation. Higher nonverbal cognitive ability was associated with higher competence in both domains, higher motivation in both domains, lower anxiety in both domains, and younger age. Finally, older children had lower math and reading competence, greater math and reading anxiety, and lower reading motivation. All of these correlations were statistically significant. The correlation between age and math motivation was not statistically significant at the 0.05 level.

### Within- and Across-Domain Socio-Emotional Characteristics Predict Math Competence

#### Predictors of Broad Mathematics

Socio-emotional characteristics in both math and reading predict math competence across both math measures. In Table 4, we present a taxonomy of models including parameter estimates, standard errors, and significance levels that illustrate the relation between Broad Mathematics, socio-emotional characteristics, and nonverbal cognitive ability. Model B1 shows the statistically significant, negative relation between Broad Mathematics and math anxiety, in which a one-point increment in math anxiety is associated with a 0.92-point decrement in Broad Mathematics score, on average. As Model B2 shows, both math anxiety and reading anxiety have statistically significant relations with Broad Mathematics, controlling for each other, in which higher anxiety predicts lower math competence, on average. Model B3 shows that when math motivation is a predictor, the relation between reading anxiety and Broad Mathematics is essentially unchanged, while the relation between math anxiety and Broad Mathematics is no longer statistically significant. In this model, math motivation has a statistically significant relation with Broad Mathematics, in which a one-point increment in math motivation predicts a 0.81-point increment in Broad Mathematics score, on average. Because math anxiety does not predict math competence when controlling for math motivation, we removed math anxiety from subsequent models in this taxonomy.

**Table 4:** Taxonomy of models showing the relation between Broad Mathematics skills and within and across domain anxiety and motivation, and nonverbal cognitive ability (*n* = 146).

	**B1**	**B2**	**B3**	**B4**	**B5**
Intercept	122.805^***^	131.118^***^	67.329^***^	51.127^***^	8.347
	(4.072)	(4.807)	(13.659)	(14.676)	(13.840)
Math anxiety	−0.918^***^	−0.680^***^	0.067		
	(0.164)	(0.178)	(0.224)		
Reading anxiety		−0.628^**^	−0.651^***^	−0.401^†^	−0.330^†^
		(0.206)	(0.191)	(0.226)	(0.193)
Math motivation			0.807^***^	0.740^***^	0.542^***^
			(0.163)	(0.121)	(0.107)
Reading motivation				0.275^†^	0.138^†^
				(0.166)	(0.143)
Nonverbal cognitive ability					0.541^***^
					(0.074)
*R* ^2^	0.179	0.229	0.342	0.354	0.532

^*^p < 0.05,

** *p < 0.01, and*

*** *p < 0.001*.

† *Reading anxiety and reading motivation jointly predict Broad Mathematics. Standard errors in parentheses*.

We next examined whether the relation between Broad Mathematics and reading anxiety and math motivation would remain when controlling for reading motivation. As Model B4 shows, reading motivation does not have a statistically significant relation with Broad Mathematics, and when controlling for reading motivation, the relation between reading anxiety and Broad Mathematics is no longer statistically significant. Due to the correlation between reading anxiety and reading motivation (Table 3), we examined whether they have a joint effect on Broad Mathematics. Using a general linear hypothesis test, we tested the null hypothesis that reading anxiety and reading motivation jointly have no effect on Broad Mathematics. We rejected the null hypothesis [*F*_(2,142)_ = 7.68, *p* = 0.0007], concluding that reading anxiety and reading motivation jointly predict Broad Mathematics.

Model B5 in Table 4 shows that relations between Broad Mathematics and socio-emotional characteristics within and across domain remain essentially unchanged when controlling for nonverbal cognitive ability. The statistically significant joint effect of reading anxiety and reading motivation was also unchanged [*F*_(2,141)_ = 4.59, *p* = 0.012]. This joint effect persisted in all subsequent models for Broad Mathematics.

In subsequent models, we did not find statistically significant interactions between nonverbal cognitive ability and math motivation [β = −0.003, *SE* = 0.007, and *p* = 0.678] or nonverbal cognitive ability and reading anxiety (β = 0.009, *SE* = 0.011, and *p* = 0.395). There were also no statistically significant main effects of age (β = −0.640, *SE* = 0.966, and *p* = 0.509) or gender (β = 3.937, *SE* = 2.067, and *p* = 0.059), controlling for socio-emotional characteristics and nonverbal cognitive ability. For the final model Model B5, we used a Shapiro–Wilk *W*-test to test the null hypothesis that the residuals from Model B5 are normally distributed in the population. We did not reject the null hypothesis (*W* = 0.989, *p* = 0.293) and concluded that there was not a violation of normality.

In sum, math motivation, the joint effect of reading anxiety and reading motivation, and nonverbal cognitive ability predict Broad Mathematics, controlling for the other predictors in the model. In Supplementary Figure 1A, we illustrate the relation between predicted Broad Mathematics and math motivation for children of lower (25th percentile) and higher (75th percentile) reading anxiety who have average nonverbal cognitive ability and reading motivation. The difference between the two lines in Supplementary Figure 1A is not statistically significant, since the joint effect of reading anxiety and reading motivation predicts Broad Mathematics. However, we illustrate this relation at higher and lower levels of math anxiety to facilitate visual comparison with the statistically significant relation in Supplementary Figure 1B.

#### Predictors of Math Fluency

In Table 5, we show a taxonomy of models of the relation of Math Fluency with socio-emotional measures of math and reading, and nonverbal cognitive ability. Model F1 shows the statistically significant negative relation with Math Fluency, in which a one-point increment in math anxiety is associated with a 0.88-point decrement in Math Fluency, on average. Model F2 shows Math Fluency has statistically significant relations with math anxiety and reading anxiety, controlling for the other. Model F3 shows a positive, statistically significant relation between Math Fluency and math motivation, controlling for math and reading anxiety. However, with the addition of math motivation, math anxiety no longer has a statistically significant relation with Math Fluency. In a subsequent model, we tested whether these relations would remain when controlling for reading motivation. Reading motivation did not predict Math Fluency (β = 0.136, *SE* = 0.171, and *p* = 0.430) and its inclusion in the model did not substantively change results from Model F3. In Model F4, we added nonverbal cognitive ability as a predictor. Relations between Math Fluency and reading anxiety, math motivation, and nonverbal cognitive ability were each statistically significant, controlling for the other predictors in the model. Subsequent models did not show statistically significant interactions between reading anxiety and math motivation (β = 0.0005, *SE* = 0.014, and *p* = 0.973), or relations between Math Fluency and age (β = −1.940, *SE* = 1.056, and *p* = 0.068) or Math Fluency and gender (β = 2.732, *SE* = 2.239, and *p* = 0.225), all else equal. A Shapiro–Wilk test of the residuals from Model F4 showed no violation of normality (*W* = 0.988, *p* = 0.225). In Supplementary Figure 1B, we illustrate the relation between predicted Math Fluency and math motivation for children of lower (25th percentile) and higher (75th percentile) reading anxiety and of average nonverbal cognitive ability. The figure shows that children with lower reading anxiety have higher Math Fluency at every level of math motivation, on average.

**Table 5:** Taxonomy of models examining the relation between Math Fluency, math and reading anxiety, math motivation, and nonverbal cognitive ability (*n* = 146).

	**F1**	**F2**	**F3**	**F4**
Intercept	119.592^***^	126.859^***^	72.867^***^	30.274^*^
	(4.052)	(4.819)	(14.024)	(11.619)
Math anxiety	−0.876^***^	−0.668^***^	−0.036	
	(0.163)	(0.178)	(0.230)	
Reading anxiety		−0.549^**^	−0.569^**^	−0.434^*^
		(0.207)	(0.196)	(0.172)
Math motivation			0.683^***^	0.533^***^
			(0.168)	(0.118)
Nonverbal cognitive ability				0.423^***^
				(0.082)
*R* ^2^	0.167	0.206	0.289	0.402

* *p < 0.05*,

** *p < 0.01, and*

*** *p < 0.001. Standard errors in parentheses*.

Taken together, analyses show that, controlling for nonverbal cognitive ability, both within- and across-domain socio-emotional characteristics predict math competence, on average.

### Within-Domain Socio-Emotional Characteristics Predict Reading Competence

#### Predictors of Total Word Reading Efficiency

Table 6 shows a taxonomy of models of the relation between Total Word Reading Efficiency (i.e., timed word and pseudoword reading) and reading anxiety, motivation to read, and nonverbal cognitive ability. Model T1 shows the statistically significant relation between Total Word Reading Efficiency and reading anxiety, in which a one-point increment in reading anxiety is associated with a 1.2-point decrement in Total Word Reading Efficiency, on average. The addition of math anxiety to the model did not yield a statistically significant relation (β = 0.015, *SE* = 0.171, and *p* = 0.929). In Model T2, we show that reading anxiety and reading motivation each predict Total Word Reading Efficiency, on average, controlling for the other. In a subsequent model, we found that math motivation did not have a statistically significant relation with Total Word Reading Efficiency, controlling for reading anxiety and reading motivation (β = 0.144, *SE* = 0.114, and *p* = 0.209). These results suggest that when considered together, within-domain anxiety and motivation predict reading competence, while across-domain anxiety and motivation do not.

**Table 6:** Taxonomy of models examining the relation between Total Word Reading Efficiency, reading anxiety, reading motivation, and nonverbal cognitive ability (*n* = 146).

	**T1**	**T2**	**T3**	**T4**
Intercept	124.423^***^	54.679^***^	17.082	−0.657
	(4.115)	(12.685)	(13.595)	(8.986)
Reading anxiety	−1.217^***^	−0.421^*^	−0.337	
	(0.178)	(0.212)	(0.195)	
Reading motivation		0.889^***^	0.758^***^	0.914^***^
		(0.155)	(0.144)	(0.113)
Nonverbal cognitive ability			0.387^***^	0.397^***^
			(0.073)	(0.073)
*R* ^2^	0.245	0.387	0.489	0.478

* *p < 0.05*,

^**^p < 0.01, and

*** *p < 0.001. Standard errors in parentheses*.

Model T3 in Table 6 shows that nonverbal cognitive ability has a statistically significant relation with Total Word Reading Efficiency, controlling for reading anxiety and reading motivation. Further, this model shows that when controlling for nonverbal cognitive ability, the relation between Total Word Reading Efficiency and reading anxiety is no longer statistically significant. We refit the model, removing reading anxiety (Model T4). In subsequent models, we did not find a statistically significant interaction between reading motivation and nonverbal cognitive ability (β = −0.0004, *SE* = 0.009, and *p* = 0.958), or main effects of age (β = −1.890, *SE* = 0.973, and *p* = 0.054) or gender (β = 2.961, *SE* = 2.016995, and *p* = 0.144). A Shapiro–Wilk test of the residuals from Model T4 showed no violation of normality (*W* = 0.993, *p* = 0.682). Supplementary Figure 1C displays predicted Total Word Reading Efficiency by reading motivation for children of average nonverbal cognitive ability.

#### Predictors of Basic Skills

Lastly, Table 7 shows a selection of models of the relation between Basic Skills and reading anxiety, reading motivation, nonverbal cognitive ability, age, and gender. Model S1 shows the negative, statistically significant relation between reading anxiety and Basic Skills. In a subsequent model, we did not find a statistically significant relation between math anxiety and Basic Skills (β = −0.048, *SE* = 0.182, and *p* = 0.792) and therefore we excluded math anxiety from subsequent models. Model S2 shows the statistically significant relation between reading motivation and Basic Skills, controlling for reading anxiety. However, controlling for reading motivation, the relation between Basic Skills and reading anxiety was no longer statistically significant. Similar to math anxiety, math motivation did not have a statistically significant relation with Basic Skills (β = 0.150, *SE* = 0.125, and *p* = 0.233). Model S3 shows that the statistically significant relation between reading motivation and Basic Skills persists when controlling for nonverbal cognitive ability. In Models S4 and S5, respectively, we add the effects of age and gender, which both have statistically significant relations with Basic Skills, all else equal. Model S5 also shows that, controlling for age, gender, and nonverbal cognitive ability, reading motivation maintains a statistically significant relation with Basic Skills. There were no statistically significant interactions (all *p*s > 0.35). A Shapiro–Wilk test of the residuals from Model S5 showed no violation of normality (*W* = 0.989, *p* = 0.307). Supplementary Figure 1D shows the relation between predicted Basic Skills and reading motivation, on average. The graph shows the 4.5-point predicted difference in Basic Skills between boys and girls of average nonverbal cognitive ability and average age.

**Table 7:** Taxonomy of models examining the relation between Basic Skills, reading anxiety, reading motivation, nonverbal cognitive ability, age, and gender (*n* = 146).

	**S1**	**S2**	**S3**	**S4**	**S5**
Intercept	123.359^***^	59.558^***^	−0.352	50.147^**^	48.818^**^
	(4.383)	(13.915)	(9.768)	(18.540)	(18.328)
Reading anxiety	−1.151^***^	−0.424			
	(0.190)	(0.233)			
Reading motivation		0.813^***^	0.817^***^	0.738^***^	0.789^***^
		(0.170)	(0.123)	(0.121)	(0.122)
Nonverbal cognitive ability			0.449^***^	0.354^***^	0.341^***^
			(0.079)	(0.082)	(0.082)
Age				−3.281^**^	−3.491^***^
				(1.036)	(1.028)
Boy					4.483^*^
					(2.119)
*R* ^2^	0.204	0.314	0.427	0.465	0.481

* *p < 0.05*,

** *p < 0.01, and*

*** *p < 0.001. Standard errors in parentheses*.

In sum, reading motivation predicted Basic Skills, controlling for nonverbal cognitive ability, age, and gender. There were no across-domain relations between Basic Skills and math socio-emotional characteristics.

## Discussion

In this study, we provide an examination of the within and across-domain relations among anxiety, motivation, and competence for math and reading for an academically-diverse group of children. We leveraged existing math anxiety and reading motivation scales to create novel parallel measures of reading anxiety and math motivation to measure socio-emotional characteristics across domains. We found within domain relations among anxiety, motivation, and competence for both math and reading. We also found a unidirectional across-domain relation between reading socio-emotional characteristics and math competence, which persisted when accounting for math motivation, nonverbal cognitive ability, age, and gender.

This study contributes to burgeoning research focused on an individual differences approach to studying children with and without learning disabilities across domains. The multiple regression approach we use facilitates the inclusion of children who do not fit into group criteria. This provides a sample that more accurately represents a continuum of learners. As Peters and Ansari (2019) discuss, an individual differences approach avoids several challenges inherent to group comparisons. Group comparisons may include arbitrary score cut-offs, which does not produce groups with “specific and separable deficits,” (p. 5). Group comparisons may also mask variation within groups and may lead to an inadequate examination of comorbidities across domains, such as math and reading. Indeed, the notion that characteristics of math and reading disabilities are related dimensions along a continuum may better describe struggling learners (Branum-Martin et al., 2013) and thus can better illuminate relations among cognitive and socio-emotional factors within and across domains.

### Socio-Emotional Predictors of Math Competence Are Within and Across Domain

We found that math anxiety correlated with both measures of math competence. However, this relation was no longer statistically significant controlling for math motivation. Rather, math motivation was a stronger predictor of math competence than math anxiety. Together with prior studies showing a reciprocal link between math anxiety and math motivation across time (Ahmed et al., 2012; Seaton et al., 2014; Gunderson et al., 2018) and math self-concept as a mediator between math anxiety and achievement (Justicia-Galiano et al., 2017), results underscore the need to attend to math motivation as an important socio-emotional predictor of mathematics skills. This in turn raises questions about how math motivation may have factored into the robust negative correlations between math anxiety and math achievement found in prior studies (for meta-analyses, see Hembree, 1990; Ma, 1999; and Barroso et al., 2021). Given the disproportionate research focus on math anxiety, results suggest the need for greater emphasis on math motivation and its interplay with math anxiety as they relate to math competence. In line with studies that have included measures of math anxiety and motivation (e.g., Lai et al., 2015; Justicia-Galiano et al., 2017), future studies should likewise include measures of both math anxiety and math motivation to more comprehensively illuminate socio-emotional factors that impact math achievement.

In line with our hypotheses, reading anxiety and reading motivation each were correlated with math competence. These socio-emotional characteristics jointly predicted math competence controlling for math motivation and nonverbal cognitive ability. This finding suggests that interrelations among math and reading domains include socio-emotional dimensions, in addition to cognitive, neural, and genetic ones (e.g., Kovas et al., 2007; Pollack and Ashby, 2018; Vanbinst et al., 2020b). Indeed, just as good reading skills may facilitate math skills, but not necessarily the reverse (Erbeli et al., 2021), how learners feel about reading may not just facilitate reading skills, but math skills as well.

We found that reading anxiety and reading motivation related to math competence differently across math outcomes. The joint effect of reading anxiety and reading motivation predicted Broad Mathematics, while reading anxiety (but not reading motivation) predicted Math Fluency, all else equal. What might account for this difference? We speculate that relations between socio-emotional characteristics of reading and math skills may be dependent on the ways in which math tasks involve reading or reading-related skills. Broad Mathematics is a measure of fact retrieval, procedural knowledge, and applications and problem solving that involve written language (Schrank et al., 2014). This broader conceptualization of math may therefore engage both reading anxiety and motivation, through the connection between written language and math problems (e.g., Lewis and Mayer, 1987; Hegarty et al., 1995; van der Schoot et al., 2009). In contrast, Math Fluency narrowly focuses on timed math fact retrieval across addition, subtraction, and multiplication (Schrank et al., 2014), and so may engage socio-emotional characteristics of reading differently. Reading anxiety, but not reading motivation, predicted Math Fluency controlling for socio-emotional characteristics for math. One potential explanation may be shared underlying mechanisms for arithmetic fact fluency and reading skills, such as retrieval fluency (Willburger et al., 2008; Koponen et al., 2020). Difficulty with retrieval fluency that may contribute to higher levels of reading anxiety may likewise relate to performance on math tasks that heavily engage retrieval, such as fact fluency, even when accounting for socio-emotional characteristics for math. Together, results suggest that across-domain relations between math competence and reading anxiety and reading motivation may vary by math task, according to associated cognitive mechanisms.

### Socio-Emotional Predictors of Reading Competence Are Within Domain

The present study adds to a nascent body of literature on the relations among reading anxiety, reading motivation, and reading competence. Across both reading outcomes, reading anxiety correlated with reading competence, but did not predict reading competence controlling for reading motivation. Rather, reading motivation predicted reading competence, controlling for nonverbal cognitive ability, age, and gender. These results align with Katzir et al. (2018), who reported more consistent relations between reading skills and reading self-concept than between reading skills and reading anxiety in children without learning disabilities. However, our results are in contrast to Ramirez et al. (2019), who found a stronger relation between reading competence and reading anxiety than reading competence and positive reading affect in children without learning disabilities. One potential reason for these differences may be that both our study and Katzir et al. (2018) included measures of reading self-concept, whereas Ramirez et al. (2019) measured positive reading affect. Additionally, we found a main effect of gender, such that boys had higher untimed reading skills than girls on average, but found no interaction between gender and reading motivation. This contrasts with related studies that have shown higher reading accuracy for girls, on average (Katzir et al., 2018) and an interaction between gender and reading anxiety and motivation (Katzir et al., 2018; Ramirez et al., 2019). Differences between samples, motivational and affective constructs, and analytic approach may account for the lack of convergence among studies. With so few studies of reading anxiety (and thus reading anxiety, motivation, and competence), additional research is needed to clarify these relations and discrepancies across studies.

Partially in line with our hypothesis, reading competence was negatively correlated with math anxiety and positively correlated with math motivation. However, these relations were not robust; neither math anxiety nor math motivation predicted reading competence when controlling for socio-emotional characteristics in reading. The correlation between math anxiety and reading competence may have been driven by the correlation of each with reading anxiety, with an analogous pattern for motivation. Similar to our speculation above, one possibility may be that timed and untimed word and pseudoword reading does not sufficiently engage math-related content to trigger math anxiety. Rather, feelings of anxiety that are associated with cognitive processes like automaticity may impact math and reading domains, and may be accounted for by reading anxiety when reading.

### Limitations and Next Steps

The correlational analyses in this study do not support causal interpretations of within or across-domain relations between socio-emotional characteristics and competence. Similarly, prior research on relations among socio-emotional characteristics and between those characteristics and competence suggests reciprocal relations (e.g., Foley et al., 2017; Gunderson et al., 2018; Ramirez et al., 2019). With one wave of data, we are unable to speak to how relations may change over time or impact students differently in other age bands. Future studies can build on the cross-sectional analyses in the present study to examine how socio-emotional characteristics may reciprocally relate to each other and interact with math and reading performance, within and across domains. In addition, future studies can test these relations cross-sectionally and longitudinally by also incorporating measures of general anxiety and motivation. Additionally, younger children in our sample had higher math and reading competence, on average, than older children, as evidenced by the zero-order correlations between competence and age. However, results show that for most outcomes, age was not a statistically significant predictor of competence and in all sets of models, the inclusion of age did not substantially change results, suggesting this relation did not drive the results.

Our results raise several considerations for future research. As one example, approaches to alleviate math anxiety have spanned cognitive therapy, task reappraisal, pre-task expressive writing, noninvasive brain stimulation, and skill improvement (for a review, see Dowker, 2019b). The stronger relation of within-domain motivation for both math and reading raises the question of whether efforts to reduce anxiety and raise competence should also incorporate the improvement of motivational factors that include self-concept and value for math or reading. As anxiety and motivation are not opposite ends of the same spectrum (Dowker et al., 2016; Dowker, 2019b), interventions that combine strategies to reduce anxiety and encourage motivation may be an avenue for future research.

The unidirectional link from reading anxiety and reading motivation to math competence may likewise have implications for interventions that target socio-emotional characteristics related to math and reading. Future research should further probe the mechanisms that underlie across-domain relations between socio-emotional characteristics for reading and math competence. In turn, such research could open the door to testing whether efforts to boost reading motivation and reduce reading anxiety may have primary effects on reading competence and secondary effects on math competence.

## Conclusion

The present study suggests that the ways in which socio-emotional factors relate to competence within and across domain vary between math and reading. There is a need for greater attention to the roles that socio-emotional factors may play across math and reading for children with and without learning disabilities. Researchers and practitioners alike know that socio-emotional characteristics like anxiety and motivation matter for learning. This study contributes to an expanded view of these relations, suggesting that connections across domain may also be important to support children with and without learning disabilities.

## Data Availability Statement

The raw data supporting the conclusions of this article will be made available by the authors, without undue reservation.

## Ethics Statement

The studies involving human participants were reviewed and approved by Committee on the Use of Humans as Experimental Subjects (COUHES) at the Massachusetts Institute of Technology. Written informed consent to participate in this study was provided by the participants' legal guardian/next of kin.

## Author Contributions

JG and JAC contributed to project conceptualization, management of funding, and project supervision, management, and organization. JG, JAC, and TC contributed to grant writing. TC, KH, and JAC created the surveys. DW, TC, and KH contributed to project set-up, organization, and management. DW and KH recruited participants. CP, DW, TC, KH, AI, KW, RR, JC, IF, AD, and NA collected data. CP and JAC conceptualized the study and managed data. CP conducted statistical analysis and wrote the manuscript. TC, JG, and JAC edited the manuscript. All authors read and approved the manuscript.

## Funding

This study was funded by National Science Foundation Division of Research on Learning (Award #1644540 to JG and JAC).

## Conflict of Interest

The authors declare that the research was conducted in the absence of any commercial or financial relationships that could be construed as a potential conflict of interest.

## Publisher's Note

All claims expressed in this article are solely those of the authors and do not necessarily represent those of their affiliated organizations, or those of the publisher, the editors and the reviewers. Any product that may be evaluated in this article, or claim that may be made by its manufacturer, is not guaranteed or endorsed by the publisher.

## Supplementary Material

Supplementary Figure 1 Predicted mathematics competence (top panel) and reading competence (bottom panel) from the regression models. **(A)** shows the relation between predicted Broad Mathematics and math motivation for participants with lower reading anxiety (25th percentile) and higher reading anxiety (75th percentile) (see Table 4, Model B5). **(B)** shows the analogous relation for predicted Math Fluency (see Table 5, Model F4). **(C)** shows the relation between predicted Total Word Reading Efficiency and reading motivation (see Table 6, Model T4). **(D)** shows the relation of predicted Basic Skills and reading motivation for boys and girls (see Table 7, Model S5). For all graphs, KBIT2 is set to the sample mean. In **(D)**, participant age is also set to the sample mean (*n* = 146).

Supplementary Table 1 Descriptive statistics for age, math and reading competence, and socio-emotional measures, by group (n = 143). Note, we exclude the three participants who had math disability only due to insufficient sample size.

## References

[B1] Ahmed W. Minnaert A. Kuyper H. van der Werf G. (2012). Reciprocal relationships between math self-concept and math anxiety. Learn. Individ. Differ. 22, 385–389. 10.1016/j.lindif.2011.12.004

[B2] Arens A. K. Marsh H. W. Pekrun R. Lichtenfeld S. Murayama K. vom Hofe R. (2017). Math self-concept, grades, and achievement test scores: Long-term reciprocal effects across five waves and three achievement tracks. J. Educ. Psychol. 109, 621–634. 10.1037/edu0000163

[B3] Ashcraft M. H. (2002). Math anxiety: personal, educational, and cognitive consequences. Curr. Dir. Psychol. Sci. 11, 181–185. 10.1111/1467-8721.00196

[B4] Barbaresi W. J. Katusic S. K. Colligan R. C. Weaver A. L. Jacobsen S. J. (2005). Math learning disorder: incidence in a population-based birth cohort, 1976-82, Rochester, Minn. Ambul. Pediatr. 5, 281–289. 10.1367/A04-209R.1 16167851

[B5] Barroso C. Ganley C. M. McGraw A. L. Geer E. A. Hart S. A. Daucourt M. C. (2021). A meta-analysis of the relation between math anxiety and math achievement. Psychol. Bull. 147, 134–168. 10.1037/bul0000307 33119346 PMC8300863

[B6] Baten E. Pixner S. Desoete A. (2019). Motivational and math anxiety perspective for mathematical learning and learning difficulties, in International Handbook of Mathematical Learning Difficulties, eds. Fritz A. Haase V. G. Räsänen P. (Cham: Springer International Publishing), 457–467.

[B7] Beilock S. L. Gunderson E. A. Ramirez G. Levine S. C. (2010). Female teachers' math anxiety affects girls' math achievement. Proc. Natl. Acad. Sci. U. S. A. 107, 1860–1863. 10.1073/pnas.0910967107 20133834 PMC2836676

[B8] Boets B. De Smedt B. (2010). Single-digit arithmetic in children with dyslexia. Dyslexia 16, 183–191. 10.1002/dys.403 20440746

[B9] Branum-Martin L. Fletcher J. M. Stuebing K. K. (2013). Classification and identification of reading and math disabilities: the special case of comorbidity. J. Learn. Disabil. 46, 490–499. 10.1177/0022219412468767 23232442 PMC3836204

[B10] Carey E. Hill F. Devine A. Szücs D. (2016). The chicken or the egg? The direction of the relationship between mathematics anxiety and mathematics performance. Front. Psychol. 6:1987. 10.3389/fpsyg.2015.01987 26779093 PMC4703847

[B11] Carroll J. M. Iles J. E. (2006). An assessment of anxiety levels in dyslexic students in higher education. Br. J. Educ. Psychol. 76, 651–662. 10.1348/000709905X66233 16953967

[B12] Carroll J. M. Maughan B. Goodman R. Meltzer H. (2005). Literacy difficulties and psychiatric disorders: evidence for comorbidity. J. Child Psychol. Psychiatry 46, 524–532. 10.1111/j.1469-7610.2004.00366.x 15845132

[B13] Casey R. Levy S. E. Brown K. Brooks-Gunn J. (1992). Impaired emotional health in children with mild reading disability. J. Dev. Behav. Pediatr. 13, 256–260. 10.1097/00004703-199208000-00003 1506463

[B14] Chapman J. W. Tunmer W. E. (1995). Development of young children's reading self-concepts: an examination of emerging subcomponents and their relationship with reading achievement. J. Educ. Psychol. 87, 154–167. 10.1037/0022-0663.87.1.154

[B15] Chapman J. W. Tunmer W. E. (2003). Reading difficulties, reading-related self-perceptions, and strategies for overcoming negative self-beliefs. Read. Writ. Q. 19, 5–24. 10.1080/10573560308205

[B16] Conlon E. G. Zimmer-Gembeck M. J. Creed P. A. Tucker M. (2006). Family history, self-perceptions, attitudes and cognitive abilities are associated with early adolescent reading skills. J. Res. Read. 29, 11–32. 10.1111/j.1467-9817.2006.00290.x

[B17] Conradi K. Jang B. G. McKenna M. C. (2014). Motivation terminology in reading research: a conceptual review. Educ. Psychol. Rev. 26, 127–164. 10.1007/s10648-013-9245-z

[B18] Cui J. Zhang Y. Wan S. Chen C. Zeng J. Zhou X. (2019). Visual form perception is fundamental for both reading comprehension and arithmetic computation. Cognition 189, 141–154. 10.1016/j.cognition.2019.03.014 30953825

[B19] De Smedt B. Boets B. (2010). Phonological processing and arithmetic fact retrieval: evidence from developmental dyslexia. Neuropsychologia 48, 3973–3981. 10.1016/j.neuropsychologia.2010.10.018 20965205

[B20] Devine A. Hill F. Carey E. Szucs D. (2018). Cognitive and emotional math problems largely dissociate: Prevalence of developmental dyscalculia and mathematics anxiety. J. Educ. Psychol. 110, 431–444. 10.1037/edu0000222

[B21] Dirks E. Spyer G. van Lieshout E. C. D. M. de Sonneville L. (2008). Prevalence of combined reading and arithmetic disabilities. J. Learn. Disabil. 41, 460–473. 10.1177/0022219408321128 18768777

[B22] Dowker A. (2019a). Math anxiety and performance, in Mathematics Anxiety: What Is Known, and What is Still Missing, eds. Mammarella I. C. Caviola S. Dowker A. (New York, NY: Routledge), 62–76.

[B23] Dowker A. (2019b). ‘Maths doesn't like me anymore': the role of attitudes and emotions – implications for helping children with their arithmetical difficulties, in Individual Differences in Arithmetic: Implications for Psychology, Neuroscience and Education (Abingdon; New York, NY: Routledge), 260–285.

[B24] Dowker A. Sarkar A. Looi C. Y. (2016). Mathematics anxiety: what have we learned in 60 years? Front. Psychol. 7:508. 10.3389/fpsyg.2016.00508 27199789 PMC4842756

[B25] Duncan G. J. Dowsett C. J. Claessens A. Magnuson K. Huston A. C. Klebanov P.. (2007). School readiness and later achievement. Dev. Psychol. 43, 1428–1446. 10.1037/0012-1649.43.6.1428 18020822

[B26] Elgendi M. M. Stewart S. H. MacKay E. J. Deacon S. H. (2021). Two aspects of psychological functioning in undergraduates with a history of reading difficulties: anxiety and self-efficacy. Ann. Dyslexia 71, 84–102. 10.1007/s11881-021-00223-3 33786751

[B27] Erbeli F. Shi Q. Campbell A. R. Hart S. A. Woltering S. (2021). Developmental dynamics between reading and math in elementary school. Dev. Sci. 24:e13004. 10.1111/desc.13004 32524716 PMC7725923

[B28] Evans T. M. Flowers D. L. Napoliello E. M. Olulade O. A. Eden G. F. (2014). The functional anatomy of single-digit arithmetic in children with developmental dyslexia. Neuroimage 101, 644–652. 10.1016/j.neuroimage.2014.07.028 25067820 PMC4167356

[B29] Foley A. E. Herts J. B. Borgonovi F. Guerriero S. Levine S. C. Beilock S. L. (2017). The math anxiety-performance link: a global phenomenon. Curr. Dir. Psychol. Sci. 26, 52–58. 10.1177/0963721416672463

[B30] Froiland J. M. Oros E. (2014). Intrinsic motivation, perceived competence and classroom engagement as longitudinal predictors of adolescent reading achievement. Educ. Psychol. 34, 119–132. 10.1080/01443410.2013.822964

[B31] Ganley C. M. McGraw A. L. (2016). The development and validation of a revised version of the math anxiety scale for young children. Front. Psychol. 7:1181. 10.3389/fpsyg.2016.01181 27605917 PMC4995220

[B32] Grills A. E. Fletcher J. M. Vaughn S. Barth A. Denton C. A. Stuebing K. K. (2014). Anxiety and response to reading intervention among first grade students. Child Youth Care Forum 43, 417–431. 10.1007/s10566-014-9244-3 25431528 PMC4241545

[B33] Grills-Taquechel A. E. Fletcher J. M. Vaughn S. R. Stuebing K. K. (2012). Anxiety and reading difficulties in early elementary school: evidence for unidirectional- or bi-directional relations? Child Psychiatry Hum. Dev. 43, 35–47. 10.1007/s10578-011-0246-1 21822734 PMC3360892

[B34] Gunderson E. A. Park D. Maloney E. A. Beilock S. L. Levine S. C. (2018). Reciprocal relations among motivational frameworks, math anxiety, and math achievement in early elementary school. J. Cogn. Dev. 19, 21–46. 10.1080/15248372.2017.1421538

[B35] Hegarty M. Mayer R. E. Monk C. A. (1995). Comprehension of arithmetic word problems: A comparison of successful and unsuccessful problem solvers. J. Educ. Psychol. 87, 18–32. 10.1037/0022-0663.87.1.18

[B36] Hembree R. (1990). The nature, effects, and relief of mathematics anxiety. J. Res. Math. Educ. 21:33. 10.2307/749455

[B37] Hill F. Mammarella I. C. Devine A. Caviola S. Passolunghi M. C. Szucs D. (2016). Maths anxiety in primary and secondary school students: Gender differences, developmental changes and anxiety specificity. Learn. Individ. Differ. 48, 45–53. 10.1016/j.lindif.2016.02.006

[B38] Hossain B. Bent S. Hendren R. (2021). The association between anxiety and academic performance in children with reading disorder: a longitudinal cohort study. Dyslexia. 27, 342–354. 10.1002/dys.1680 33733531

[B39] Jain S. Dowson M. (2009). Mathematics anxiety as a function of multidimensional self-regulation and self-efficacy. Contemp. Educ. Psychol. 34, 240–249. 10.1016/j.cedpsych.2009.05.004

[B40] Jalongo M. R. Hirsh R. A. (2010). Understanding reading anxiety: new insights from neuroscience. Early Child. Educ. J. 37, 431–435. 10.1007/s10643-010-0381-5

[B41] Jameson M. M. (2014). Contextual factors related to math anxiety in second-grade children. J. Exp. Educ. 82, 518–536. 10.1080/00220973.2013.813367

[B42] Jansen B. R. J. Louwerse J. Straatemeier M. Van der Ven S. H. G. Klinkenberg S. Van der Maas H. L. J. (2013). The influence of experiencing success in math on math anxiety, perceived math competence, and math performance. Learn. Individ. Differ. 24, 190–197. 10.1016/j.lindif.2012.12.014

[B43] Justicia-Galiano M. J. Martín-Puga M. E. Linares R. Pelegrina S. (2017). Math anxiety and math performance in children: the mediating roles of working memory and math self-concept. Br. J. Educ. Psychol. 87, 573–589. 10.1111/bjep.12165 28561304

[B44] Katzir T. Kim Y.-S. G. Dotan S. (2018). Reading self-concept and reading anxiety in second grade children: the roles of word reading, emergent literacy skills, working memory and gender. Front. Psychol. 9:1180. 10.3389/fpsyg.2018.01180 30050483 PMC6050408

[B45] Kaufman A. S. Kaufman N. L. (2004). Kaufman Brief Intelligence Test Second Edition (KBIT-2). Pearson. Available online at: https://www.pearsonassessments.com/store/usassessments/en/Store/Professional-Assessments/Cognition-%26-Neuro/Non-Verbal-Ability/Kaufman-Brief-Intelligence-Test-%7C-Second-Edition/p/100000390.html (accessed March 27, 2021).

[B46] Kersey A. J. Wakim K.-M. Li R. Cantlon J. F. (2019). Developing, mature, and unique functions of the child's brain in reading and mathematics. Dev. Cogn. Neurosci. 39:100684. 10.1016/j.dcn.2019.100684 31398551 PMC6886692

[B47] Koerte I. K. Willems A. Muehlmann M. Moll K. Cornell S. Pixner S.. (2016). Mathematical abilities in dyslexic children: a diffusion tensor imaging study. Brain Imaging Behav. 10, 781–791. 10.1007/s11682-015-9436-y 26286825

[B48] Koponen T. Eklund K. Heikkil,ä R. Salminen J. Fuchs L. Fuchs D.. (2020). Cognitive correlates of the covariance in reading and arithmetic fluency: importance of serial retrieval fluency. Child Dev. 91, 1063–1080. 10.1111/cdev.13287 31292957

[B49] Kovas Y. Haworth C. M. A. Harlaar N. Petrill S. A. Dale P. S. Plomin R. (2007). Overlap and specificity of genetic and environmental influences on mathematics and reading disability in 10-year-old twins. J. Child Psychol. Psychiatry 48, 914–922. 10.1111/j.1469-7610.2007.01748.x 17714376 PMC2694440

[B50] Kriegbaum K. Jansen M. Spinath B. (2015). Motivation: a predictor of PISA's mathematical competence beyond intelligence and prior test achievement. Learn. Individ. Differ. 43, 140–148. 10.1016/j.lindif.2015.08.026

[B51] Krinzinger H. Kaufmann L. Willmes K. (2009). Math anxiety and math ability in early primary school years. J. Psychoeduc. Assess. 27, 206–225. 10.1177/0734282908330583 20401159 PMC2853710

[B52] Lai Y. Zhu X. Chen Y. Li Y. (2015). Effects of mathematics anxiety and mathematical metacognition on word problem solving in children with and without mathematical learning difficulties. PLoS One 10:e0130570. 10.1371/journal.pone.0130570 26090806 PMC4474805

[B53] Landerl K. Moll K. (2010). Comorbidity of learning disorders: prevalence and familial transmission. J. Child Psychol. Psychiatry 51, 287–294. 10.1111/j.1469-7610.2009.02164.x 19788550

[B54] Lewis A. B. Mayer R. E. (1987). Students' miscomprehension of relational statements in arithmetic word problems. J. Educ. Psychol. 79, 363–371. 10.1037/0022-0663.79.4.363

[B55] Lohbeck A. (2018). Self-concept and self-determination theory: math self-concept, motivation, and grades in elementary school children. Early Child Dev. Care 188, 1031–1044. 10.1080/03004430.2016.124177820447334

[B56] Luttenberger S. Wimmer S. Paechter M. (2018). Spotlight on math anxiety. Psychol. Res. Behav. Manag. 11, 311–322. 10.2147/PRBM.S141421 30123014 PMC6087017

[B57] Ma X. (1999). A meta-analysis of the relationship between anxiety toward mathematics and achievement in mathematics. J. Res. Math. Educ. 30, 520–540. 10.2307/749772

[B58] Malloy J. A. Marinak B. A. Gambrell L. B. Mazzoni S. A. (2013). Assessing motivation to read: the motivation to read profile-revised. Read. Teach. 67, 273–282. 10.1002/trtr.1215

[B59] Peters L. Ansari D. (2019). Are specific learning disorders truly specific, and are they disorders? Trends Neurosci. Educ. 17:100115. 10.1016/j.tine.2019.100115 31685130

[B60] Piccolo L. R. Giacomoni C. H. Julio-Costa A. Oliveira S. Zbornik J. Haase V. G.. (2017). Reading anxiety in L1: reviewing the concept. Early Child. Educ. J. 45, 537–543. 10.1007/s10643-016-0822-x

[B61] Pollack C. Ashby N. C. (2018). Where arithmetic and phonology meet: the meta-analytic convergence of arithmetic and phonological processing in the brain. Dev. Cogn. Neurosci. 30, 251–264. 10.1016/j.dcn.2017.05.003 28533112 PMC6969128

[B62] Psychological Corporation (2009). WIAT III: Wechsler Individual Achievement Test. San Antonio, TX.: Psychological Corp.

[B63] Punaro L. Reeve R. (2012). Relationships between 9-year-olds' math and literacy worries and academic abilities. Child Dev. Res. (2012). 2012:e359089. 10.1155/2012/359089

[B64] Ramirez G. Fries L. Gunderson E. Schaeffer M. W. Maloney E. A. Beilock S. L.. (2019). Reading anxiety: an early affective impediment to children's success in reading. J. Cogn. Dev. 20, 15–34. 10.1080/15248372.2018.1526175

[B65] Ramirez G. Shaw S. T. Maloney E. A. (2018). Math anxiety: past research, promising interventions, and a New Interpretation Framework. Educ. Psychol. 53, 145–164. 10.1080/00461520.2018.1447384

[B66] Retelsdorf J. Köller O. Möller J. (2011). On the effects of motivation on reading performance growth in secondary school. Learn. Instr. 21, 550–559. 10.1016/j.learninstruc.2010.11.001

[B67] Rubinsten O. Tannock R. (2010). Mathematics anxiety in children with developmental dyscalculia. Behav. Brain Funct. 6:46. 10.1186/1744-9081-6-46 20633269 PMC2913999

[B68] Schrank F. A. Mather N. McGrew K. S. (2014). Woodcock-Johnson IV Tests of Achievement. Rolling Meadows, IL: Riverside Publishing.

[B69] Seaton M. Parker P. Marsh H. W. Craven R. G. Yeung A. S. (2014). The reciprocal relations between self-concept, motivation and achievement: juxtaposing academic self-concept and achievement goal orientations for mathematics success. Educ. Psychol. 34, 49–72. 10.1080/01443410.2013.825232

[B70] Simmons F. R. Singleton C. (2008). Do weak phonological representations impact on arithmetic development? A review of research into arithmetic and dyslexia. Dyslexia Chichester Engl. 14, 77–94. 10.1002/dys.341 17659647

[B71] Suárez-Pellicioni M. Núñez-Peña M. I. Colomé À. (2016). Math anxiety: a review of its cognitive consequences, psychophysiological correlates, and brain bases. Cogn. Affect. Behav. Neurosci. 16, 3–22. 10.3758/s13415-015-0370-7 26250692

[B72] Szczygieł M. Pieronkiewicz B. (2021). Exploring the nature of math anxiety in young children: Intensity, prevalence, reasons. Math. Think. Learn. 1–19. 10.1080/10986065.2021.1882363

[B73] Torgesen J. K. Rashotte C. A. Wagner R. K. (2012). TOWRE 2: Test of Word Reading Efficiency. Available online at: https://www.proedinc.com/Products/13910/towre2-test-of-word-reading-efficiencysecond-edition-complete-kit.aspx (accessed January 3, 2021).

[B74] Urdan T. Schoenfelder E. (2006). Classroom effects on student motivation: goal structures, social relationships, and competence beliefs. J. Sch. Psychol. 44, 331–349. 10.1016/j.jsp.2006.04.003

[B75] van der Schoot M. Bakker Arkema A. H. Horsley T. M. van Lieshout E. C. D. M. (2009). The consistency effect depends on markedness in less successful but not successful problem solvers: an eye movement study in primary school children. Contemp. Educ. Psychol. 34, 58–66. 10.1016/j.cedpsych.2008.07.002

[B76] Vanbinst K. Bellon E. Dowker A. (2020a). Mathematics anxiety: an intergenerational approach. Front. Psychol. 11:1648. 10.3389/fpsyg.2020.01648 32793047 PMC7385133

[B77] Vanbinst K. van Bergen E. Ghesquière P. De Smedt B. (2020b). Cross-domain associations of key cognitive correlates of early reading and early arithmetic in 5-year-olds. Early Child. Res. Q. 51, 144–152. 10.1016/j.ecresq.2019.10.009

[B78] Vukovic R. K. Lesaux N. K. Siegel L. S. (2010). The mathematics skills of children with reading difficulties. Learn. Individ. Differ. 20, 639–643. 10.1016/j.lindif.2010.08.00431105385

[B79] Wang Z. Lukowski S. L. Hart S. A. Lyons I. M. Thompson L. A. Kovas Y.. (2015). Is Math Anxiety Always Bad for Math Learning? The Role of Math Motivation. Psychol. Sci. 26, 1863–1876. 10.1177/0956797615602471 26518438 PMC4679544

[B80] Willburger E. Fussenegger B. Moll K. Wood G. Landerl K. (2008). Naming speed in dyslexia and dyscalculia. Learn. Individ. Differ. 18, 224–236. 10.1016/j.lindif.2008.01.003

[B81] Woodcock R. W. (2011). Woodcock Reading Mastery Test, 3rd Edn. Bloomington, MN: Pearson.

[B82] Wu S. S. Barth M. Amin H. Malcarne V. Menon V. (2012). Math anxiety in second and third graders and its relation to mathematics achievement. Front. Psychol. 3:162. 10.3389/fpsyg.2012.00162 22701105 PMC3369194

[B83] Zakaria E. Nordin N. M. (2008). The effects of mathematics anxiety on matriculation students as related to motivation and achievement. Eurasia J. Math. Sci. Technol. Educ. 4, 27–30. 10.12973/ejmste/75303

