# Supplementary figures and images for: Anxiety, Motivation, and Competence in Mathematics and Reading for Children With and Without Learning Difficulties
Source: Front Psychol. 2021 Oct 7;12:704821. doi: 10.3389/fpsyg.2021.704821 (PMC8528962; doi:10.3389/fpsyg.2021.704821)

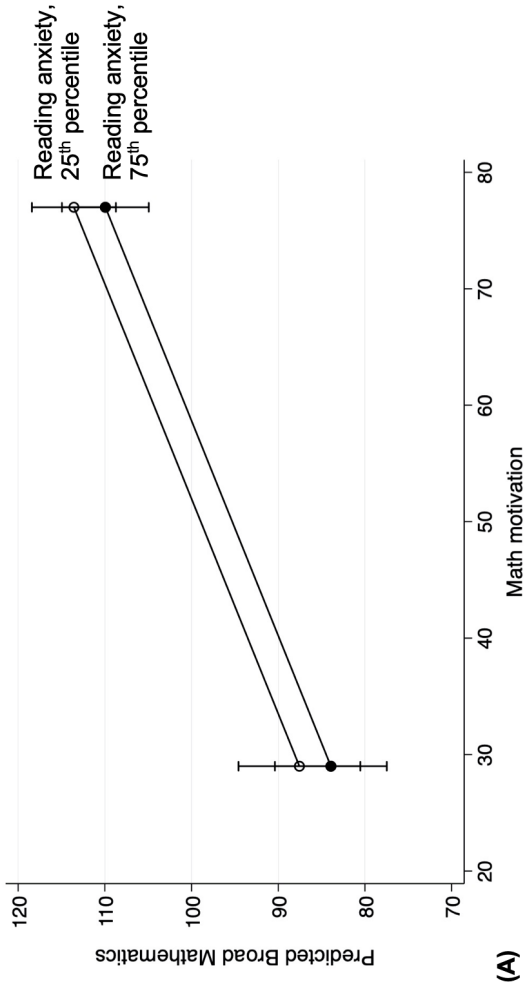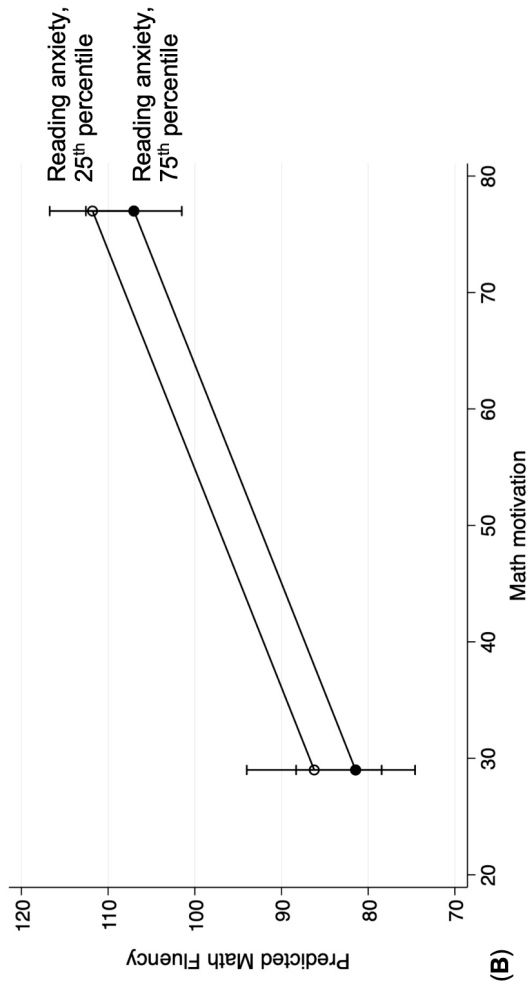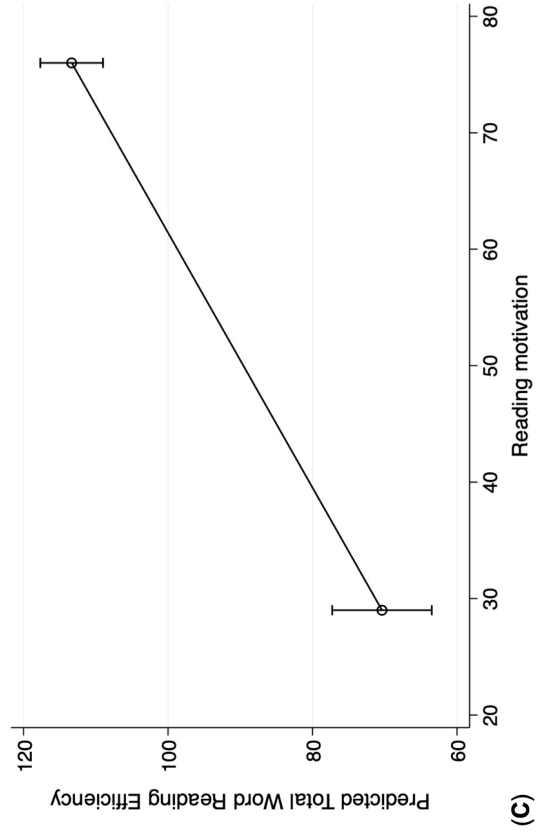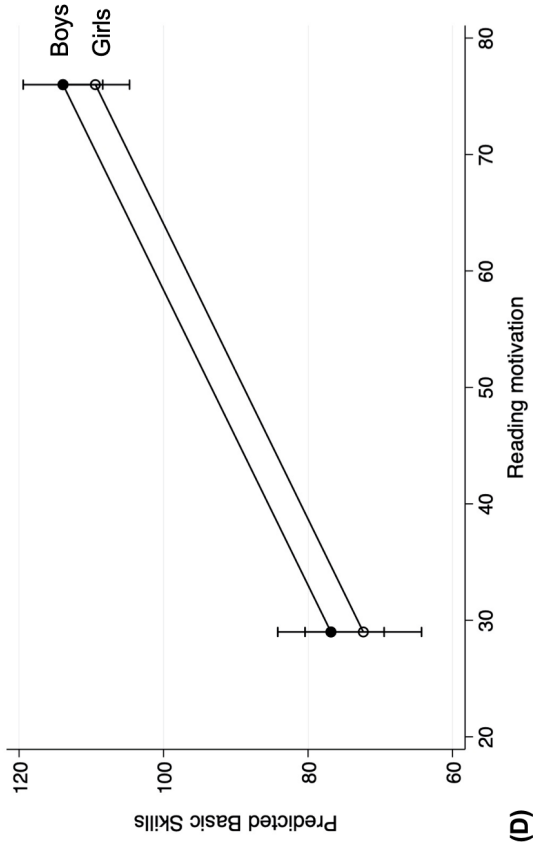

Supplement: Supplementary Figure 1 — Predicted mathematics competence (top panel) and reading competence (bottom panel) from the regression models. (A) shows the relation between predicted Broad Mathematics and math motivation for participants with lower reading anxiety (25th percentile) and higher reading anxiety (75th percentile) (see Table 4, Model B5). (B) shows the analogous relation for predicted Math Fluency (see Table 5, Model F4). (C) shows the relation between predicted Total Word Reading Efficiency and reading motivation (see Table 6, Model T4). (D) shows the relation of predicted Basic Skills and reading motivation for boys and girls (see Table 7, Model S5). For all graphs, KBIT2 is set to the sample mean. In (D), participant age is also set to the sample mean (n = 146). [file Image_1.pdf]
