# Supplementary material for: Anxiety, Motivation, and Competence in Mathematics and Reading for Children With and Without Learning Difficulties
Source: Front Psychol. 2021 Oct 7;12:704821. doi: 10.3389/fpsyg.2021.704821 (PMC8528962; doi:10.3389/fpsyg.2021.704821)
Supplement: Supplementary Table 1 — Descriptive statistics for age, math and reading competence, and socio-emotional measures, by group (n = 143). Note, we exclude the three participants who had math disability only due to insufficient sample size. [file Table_1.docx]

Supplementary Table 1. Descriptive statistics for age, math and reading competence, and socio-emotional measures, by group (*n* = 143). Note, we exclude the three participants who had math disability only due to insufficient sample size.

|  | Typically-developing | | | Reading disability | | | Math & reading disability | | | Heterogeneous | | | |
| --- | --- | --- | --- | --- | --- | --- | --- | --- | --- | --- | --- | --- | --- |
|  | (*n* = 68) | | | (*n* = 13) | | | (*n* = 33) | | | | (*n* = 29) | | |
|  | M  (SD) | Min | Max | M  (SD) | Min | Max | M  (SD) | Min | Max | | M  (SD) | Min | Max |
| Age | 10.21 | 8.08 | 12.74 | 11.29 | 10.42 | 11.85 | 11.45 | 10.33 | 13.16 | | 10.98 | 8.25 | 12.93 |
|  | (1.15) |  |  | (0.45) |  |  | (0.77) |  |  | | (1.07) |  |  |
| Nonverbal cognitive ability | 118.68 | 92 | 143 | 105.62 | 84 | 119 | 102.22 | 82 | 131 | | 112.62 | 84 | 134 |
|  | (12.69) |  |  | (12.41) |  |  | (11.91) |  |  | | (12.78) |  |  |
| Competence |  |  |  |  |  |  |  |  |  | |  |  |  |
| Broad Mathematics | 112.03 | 94 | 136 | 102.23 | 94 | 111 | 79.97 | 58 | 98 | | 101.21 | 58 | 139 |
|  | (9.73) |  |  | (3.98) |  |  | (10.89) |  |  | | (15.46) |  |  |
| Math Fluency | 109.52 | 90 | 142 | 100.23 | 88 | 114 | 78.64 | 62 | 91 | | 97.79 | 65 | 128 |
|  | (11.90) |  |  | (7.83) |  |  | (8.82) |  |  | | (14.04) |  |  |
| Total Word Reading Efficiency | 109.43 | 91 | 130 | 85.08 | 72 | 94 | 78.61 | 64 | 104 | | 96.00 | 58 | 130 |
|  | (8.90) |  |  | (6.37) |  |  | (7.82) |  |  | | (16.50) |  |  |
| Basic Skills | 110.72 | 94 | 136 | 84.69 | 63 | 98 | 79.67 | 59 | 94 | | 93.79 | 55 | 120 |
|  | (9.39) |  |  | (9.32) |  |  | (7.51) |  |  | | (17.02) |  |  |
| Anxiety and Motivation |  |  |  |  |  |  |  |  |  | |  |  |  |
| Math anxiety | 21.03 | 13 | 39 | 24.08 | 14 | 46 | 27.09 | 15 | 47 | | 24.24 | 13 | 38 |
|  | (5.82) |  |  | (10.35) |  |  | (7.70) |  |  | | (6.81) |  |  |
| Math motivation | 60.53 | 43 | 74 | 58.15 | 41 | 71 | 50.85 | 29 | 70 | | 60.38 | 44 | 77 |
|  | (7.53) |  |  | (9.71) |  |  | (10.61) |  |  | | (8.63) |  |  |
| Reading anxiety | 20.04 | 13 | 44 | 22.92 | 16 | 34 | 27.03 | 13 | 41 | | 21.72 | 13 | 36 |
|  | (5.43) |  |  | (5.14) |  |  | (7.41) |  |  | | (6.45) |  |  |
| Reading motivation | 61.75 | 44 | 76 | 53.31 | 29 | 66 | 51.58 | 36 | 68 | | 61.03 | 44 | 76 |
|  | (6.72) |  |  | (9.99) |  |  | (9.20) |  |  | | (8.43) |  |  |
